# Impact and predictive modeling of risk factors for involuntary psychiatric admissions before and during COVID-19: insights from a Romanian tertiary hospital

**DOI:** 10.3389/fpubh.2025.1624219

**Published:** 2025-09-08

**Authors:** Ciprian Ionuț Băcilă, Monica Cornea, Bogdan Ioan Vintilă, Andrei Lomnasan, Adrian Gheorghe Boicean, Andreea Maria Grama, Claudiu Matei, Bogdan Neamtu

**Affiliations:** ^1^Neuroscience Scientific Research Group “Dr. Gheorghe Preda” Clinical Hospital Psychiatry of Sibiu, Sibiu, Romania; ^2^Faculty of Medicine, Lucian Blaga University of Sibiu, Sibiu, Romania; ^3^County Clinical Emergency Hospital of Sibiu, Sibiu, Romania; ^4^Medlife Polisano Hospital, Sibiu, Romania; ^5^Center for Research in Mathematics and Applications, “Lucian Blaga” University of Sibiu, Sibiu, Romania; ^6^Pediatric Clinical Hospital, Sibiu, Romania; ^7^Whiting School of Engineering, Johns Hopkins University, Baltimore, MD, United States

**Keywords:** involuntary admission, psychiatric hospitalization, COVID-19 pandemic, risk factors, mental health crisis, logistic regression model, predictive analysis

## Abstract

**Introduction:**

The COVID-19 pandemic significantly reshaped involuntary psychiatric hospitalizations, disrupting the balance between patient rights, public safety, and healthcare delivery. This study aims to examine the pandemic’s impact on involuntary admissions (IA) from a major psychiatric hospital in Sibiu Romania. Furthermore, it proposes a prediction model for informed consent refusal rates (ICRR).

**Materials and methods:**

We conducted a retrospective, observational analysis of 781 involuntary admissions using records by comparing socio-demographic, clinical, and procedural variables across two periods: pre-pandemic (March 2018–February 2020) and during the pandemic (March 2020–March 2022). Variables analyzed included demographics, clinical symptoms, procedural circumstances, and hospitalization duration with Chi-Square, Cochran–Mantel–Haenszel (CMH), Breslow-Day, Cramer’s V tests and logistic regression model applied as appropriate.

**Results:**

Psychomotor agitation, aggression, and suicidal behavior were leading reasons for involuntary admission. Confirmation rates were significantly higher among non-aggressive patients (*p* < 0.0001). Schizophrenia spectrum disorders were predominant diagnoses, with significantly higher confirmation rates during the pandemic (*p* < 0.0001). Police-initiated admissions increased significantly, while family-initiated admissions significantly declined (*p* < 0.001). Other consistently significant predictors included insurance status, marital status, residence type, psychotic symptoms, psychiatric comorbidities, and the source initiating the involuntary admission request (all CMH tests *p* ≤ 0.002). Logistic regression modeling demonstrated strong predictive performance (AUC = 0.807, accuracy = 80.7%), identifying education level, alcohol consumption, psychoactive substance use, and police involvement as significant predictors of ICRR.

**Conclusion:**

The pandemic introduced significant procedural and management challenges to involuntary admissions at a tertiary hospital in Romania. Our predictive modeling highlights key factors influencing hospitalization outcomes, underscoring the critical need for streamlined ethical and procedural frameworks, strengthened multidisciplinary collaboration, and the integration of machine learning methodologies to enhance predictive accuracy and clinical decision-making in future public health crises.

## Introduction

Declared in March 2020 by the World Health Organization, the COVID-19 pandemic led to over 776 million confirmed cases (1–3) and, within its first year, triggered significant increases in major depressive and anxiety disorders globally (4, 5). In the United Kingdom, for instance, isolation and lockdown measures drove an eight-fold rise in depression, while patients hospitalized for more than 7 days with COVID-19 showed particularly high levels of depressive and anxiety symptoms (6). Nevertheless, across Europe (Germany, Italy, Portugal) and globally (South Korea, Australia), psychiatric admissions generally declined, largely due to infection fears, stricter admission criteria, reduced bed availability, and the shift to telepsychiatry (7). Beyond these changes in admission rates, the pandemic also revealed complex ethical and legal challenges in psychiatric care despite universal rights for individuals with mental illness (8). Consequently dilemmas around patient autonomy in treatment and hospitalization (9), and stigma or discrimination delayed access until emergency IA becomes necessary (10–13). IA, while legally and ethically complex, serves as a protective intervention aimed at safeguarding both the individual and society and restoring the patient’s decision-making capacity. IA requires coordinated efforts between psychiatrists and legal professionals (14–16).

Notably, international variations in IA rates or, more specifically ICRR are shaped more by legal frameworks than clinical factors (17–20). This highlights the need for detailed socio-demographic and clinical profiling to inform public-awareness strategies and uphold compliance with European human rights standards (21, 22). In this context, the COVID-19 crisis exposed both systemic vulnerabilities and areas of resilience: while partial quarantine measures reduced emergency psychiatric admissions (23–28) acute IA increased during the first lockdown period (29, 30). In Romania, IA is governed by the Mental Health Law, which mandates a psychiatrist’s evaluation of imminent danger or potential severe deterioration after exhausting voluntary options. This is followed by judicial oversight consisting of committee review within 48 h, court confirmation, monthly reassessments, and court-authorized discharge (18, 31–33) (see Appendix A).

Although some reports exist, the impact of COVID-19 on involuntary psychiatric hospitalizations in Romania and across the Balkan region remains underexplored (7). Available data, mainly from Croatia and Romania, focus primarily on overall psychiatric admissions rather than IA cases. In Croatia, a 28% decline in total psychiatric admissions was reported at the University Hospital of Split, alongside a 20% reduction in bed capacity, with a similar decrease observed at Zagreb’s Vrapče Hospital. At our institution, “Dr Gheorghe Preda” Hospital in Sibiu, previous findings showed a drop in total hospitalizations during the pandemic, accompanied by an increased average length of stay, which altered the diversity of cases managed (34).

To address the gap in understanding ICRR in Romania and at a regional level, our study analyzes the socio-demographic, clinical, and procedural factors influencing IA by comparing the pre-pandemic and pandemic periods. We hypothesized that the COVID-19 pandemic significantly altered the socio-demographic, clinical, and procedural predictors of ICRR in Romania, and that these variables can be identified through logistic regression modeling. Potential predictors were systematically selected using comprehensive statistical methods and subsequently fed into the model. While recent studies have applied machine learning techniques such as Random Forest and XGBoost, achieving AUCs between 0.68 and 0.84 (35, 36), these approaches often entail complex implementation, extensive hyperparameter tuning, and limited interpretability. In contrast, logistic regression offers a transparent, reproducible framework suited to direct clinical application, combining robust predictive performance with mathematical clarity. Given the scarcity of ML research in involuntary psychiatric admissions, our data-driven approach aims to enhance early identification of high-risk patients and improve preparedness for future public-health crises.

## Materials and methods

This is a secondary analysis in a retrospective study conducted over a period of 4 years at “Dr Gheorghe Preda” Clinical Hospital of Psychiatry from Sibiu. According to data published by the National Institute of Public Health (NIPH) (37), the hospital serves over 12,000 mentally disordered people. It is a tertiary hospital with a capacity of 453 beds and the following organizational structure: a male psychosis ward (55 beds), a female psychosis ward (63 beds), an organic mental disorders and personality disorders ward (55 beds), a gerontopsychiatry ward (50 beds), an addiction-related disorders ward (50 beds) and a chronic disorders ward (90 beds). We compared a range of factors among patients proposed for involuntary admission across two distinct timeframes: the pre-pandemic period (March 2018–February 2020) and the pandemic period (March 2020–March 2022). These factors included socio-demographic and economic characteristics, clinical and psychiatric symptoms, behavioral indicators and substance use, legal and admission circumstances, as well as restraint-related and timing variables. Our analysis focused specifically on the ICRR. The approach enabled a more nuanced understanding of the determinants influencing the confirmation of such measures and underscored the importance of identifying predictors that shape decision-making in involuntary hospitalization. Additionally, we aimed to develop and validate a machine learning model to predict the likelihood of ICRR, leveraging the comprehensive dataset derived from the examined variables.

### Research design

Inclusion criteria consisted of patients proposed for IA, *N* = 787 (either emergency room presentation or initially voluntary admission with subsequent unfavorable outcome requiring initiation of IA measure and patients aged >18). We excluded from the study all records in electronic or physical format containing insufficient and/or inconclusive data. For individuals with multiple admissions, each IA episode was included in the statistical analysis. Every hospitalization was treated as a distinct case, accounting for the specific clinical characteristics and symptomatology present at the time of admission.

### Data collection process

Data was collected from electronic records and the hospital’s IA registers in physical format, some provided by the hospital’s statistical service. Given the study’s retrospective nature and data anonymization, patients were not required to provide informed consent to participate. The socio-demographic variables considered in the analysis included age, gender, residential background, marital status, educational attainment, employment status, and insurance coverage. Clinical variables consisted of primary psychiatric diagnosis at the time of admission, presence of psychiatric comorbidities, history of relapses, time of admission (day vs. night), presence of psychotic symptoms (e.g., hallucinations or delusions noted at admission), self-aggressive behavior (including self-injurious ideation or attempts), aggressive behavior toward others, neurotoxic substance use (e.g., alcohol or psychoactive substances), and the number of hospitalization days. Additional variables analyzed comprised the application of mechanical restraint, police involvement in patient transport, the initiators of the involuntary admission (IA) request (e.g., family members, healthcare professionals, or law enforcement), whether informed consent was signed or refused by the patient during the IA committee evaluation (*informed consent refusal*), and time intervals associated with the various stages of the IA procedure. Psychiatric diagnoses were categorized according to DSM-5 and ICD-10 criteria (38, 39) as follows: organic mental disorders, including cognitive disorders (F00–F09); mental and behavioral disorders due to psychoactive substance use (F10–F19); schizophrenia, schizotypal, and delusional disorders (F20–F29); bipolar affective disorders (F30–F31); other affective disorders (F32–F39); personality and behavioral disorders (F60–F69); and other mental disorders (F40–F48.9, F50–F59, F70–F79, F80–F89, F90–F99).

### Ethical issues of research

The study was conducted according to the ethical principles stipulated in the Declaration of Helsinki (40), having prior approval from the ethics committee (No. 5381/21.04.2023). Due to the study’s retrospective nature and the complete anonymization of the data included in the analysis, informed consent from the patients was unnecessary.

### Statistical analysis

To systematically explore the factors associated with ICRR across pandemic phases, a structured multi-step statistical approach was implemented to assess both the strength and stability of associations prior to predictive modeling. Categorical variables were evaluated using chi-square tests to assess their association with ICRR across the pre-pandemic and pandemic periods, applying a significance threshold of *p* < 0.05. This criterion was consistently used across all statistical tests and models. Association strength was quantified using Cramér’s V, while the Cochran–Mantel–Haenszel (CMH) test assessed the stability of associations over time by controlling for study period. The Breslow–Day test was used to evaluate the homogeneity of odds ratios between the two-time frames.

Predictors were retained for multivariable modeling if they demonstrated at least a moderate association (Cramér’s V ≥ 0.20 in either period) or a significant CMH trend (*α* = 0.05). To address potential multicollinearity, variance inflation factors (VIF) were calculated, and only variables with VIF < 4 were included in the initial model.

A logistic regression framework was used to identify independent predictors of ICRR. Given the presence of small sample sizes and rare events in some categories, Firth’s penalized likelihood approach was applied to improve estimate stability and reduce bias. Model performance was assessed using the Area Under the Curve (AUC) from receiver operating characteristic (ROC) analysis and the Likelihood Ratio Chi-square test. Results were presented as adjusted odds ratios (ORs) with 95% confidence intervals (CIs). All analyses were conducted in SAS Studio.

## Results

During the 4-year study period, 781 people (623 patients with unique id) were admitted involuntarily out of 21,069 hospitalized in our service. In the pre-pandemic period, 24.65% of admissions were through the emergency room, and 3% were involuntary. Compared with the pre-pandemic period, during the pandemic period (March 2020–March 2022), the total number of admissions decreased significantly, but the proportion of involuntary admissions reached 5.16% of all admissions. Of the 781 cases with involuntary hospitalization included in the study, only 426 required court documentation to initiate involuntary hospitalization procedures for those who refused informed consent. In 355 cases, patients subsequently signed informed consent.

### Sociodemographic and economic factors

#### Gender and ICRR

The distribution of gender in IA differed significantly between the pandemic and pre-pandemic periods. Males became less likely to refuse consent during the pandemic, whereas females exhibited a modest but significant increase in refusal rates (Table 1). While the proportion of male admissions decreased (65.02% [*n* = 277] to 61.97% [*n* = 220]), their consent refusal rates also reduced (51.26% [*n* = 142] to 46.82% [*n* = 103]). In contrast, female admissions increased (34.98% [*n* = 149] to 38.03% [*n* = 135]), but their likelihood of refusing consent also rose (59.73% [*n* = 89] to 68.15% [*n* = 92]). While the pre-pandemic association was weak and not statistically significant (Chi-Square *p* = 0.0943, Cramer’s V = 0.0811), the pandemic period showed a statistically significant association (Chi-Square *p* < 0.0001, Cramer’s V = 0.2081), suggesting a small to moderate effect size. The relationship between gender and refusal probability remained proportionally similar (stable, Breslow-Day test *p* = 0.0767), even though refusal rates shifted (CMH test *p* = 0.0001).

**Table 1:** Distribution of informed consent refusal rates (ICRR) by socio-demographic characteristics across the pre-pandemic and pandemic periods.

Demographics and socioeconomic factors	Group study						Total
	Pre-pandemic			Pandemic			
	Informed consent		Total	Informed consent		Total	
	Signed	Refused		Signed	Refused		
	Count^as^ (%)^as^	Count^ar^ (%)^ar^	Count^a^ (%)^a^	Count^bs^ (%)^bs^	Count^br^ (%)^br^	Count^b^ (%)^b^	Count^c^ (%)^c^
Gender							
Male	135 (69.23%)	142 (61.47%)	277 (65.02%)	117 (73.13%)	103 (52.82%)	220 (61.97%)	497 (63.64%)
Female	60 (30.77%)	89 (38.53%)	149 (34.98%)	43 (26.87%)	92 (47.18%)	135 (38.03%)	284 (36.36%)
Age group							
≤20	6 (3.08%)	4 (1.73%)	10 (2.35%)	7 (4.38%)	4 (2.05%)	11 (3.10%)	21 (2.69%)
20–29	35 (17.95%)	55 (23.81%)	90 (21.13%)	28 (17.50%)	39 (20.00%)	67 (18.87%)	157 (20.10%)
30–39	43 (22.05%)	61 (26.41%)	104 (24.41%)	28 (17.50%)	40 (20.51%)	68 (19.15%)	172 (22.02%)
40–49	45 (23.08%)	52 (22.51%)	97 (22.77%)	41 (25.63%)	39 (20.00%)	80 (22.54%)	177 (22.66%)
50–59	33 (16.92%)	20 (8.66%)	53 (12.44%)	30 (18.75%)	44 (22.56%)	74 (20.85%)	127 (16.26%)
60–69	22 (11.28%)	31 (13.42%)	53 (12.44%)	15 (9.38%)	20 (10.26%)	35 (9.86%)	88 (11.27%)
≥70	11 (5.64%)	8 (3.46%)	19 (4.46%)	11 (6.88%)	9 (4.62%)	20 (5.63%)	39 (4.99%)
Marital status							
Single	50 (25.64%)	60 (25.97%)	110 (25.82%)	31 (19.38%)	49 (25.13%)	80 (22.54%)	190 (24.33%)
Married	34 (17.44%)	54 (23.38%)	88 (20.66%)	29 (18.13%)	45 (23.08%)	74 (20.85%)	162 (20.74%)
Divorced	16 (8.21%)	28 (12.12%)	44 (10.33%)	7 (4.38%)	19 (9.74%)	26 (7.32%)	70 (8.96%)
Widowed	8 (4.10%)	12 (5.19%)	20 (4.69%)	4 (2.50%)	7 (3.59%)	11 (3.10%)	31 (3.97%)
Not specified	87 (44.62%)	77 (33.33%)	164 (38.50%)	89 (55.63%)	75 (38.46%)	164 (46.20%)	328 (42.00%)
Education level							
No formal education	17 (8.72%)	2 (0.87%)	19 (4.46%)	19 (11.88%)	12 (6.15%)	31 (8.73%)	50 (6.40%)
Primary education	49 (25.13%)	49 (21.21%)	98 (23.00%)	45 (28.13%)	35 (17.95%)	80 (22.54%)	178 (22.79%)
Middle school education	44 (22.56%)	49 (21.21%)	93 (21.83%)	25 (15.63%)	34 (17.44%)	59 (16.62%)	152 (19.46%)
High school education	25 (12.82%)	64 (27.71%)	89 (20.89%)	24 (15.00)	48 (24.62%)	72 (20.28%)	161 (20.61%)
Vocational education	31 (15.90%)	24 (10.39%)	55 (12.91%)	23 (14.38%)	15 (7.69%)	38 (10.70%)	93 (1.91%)
Higher education	16 (8.21%)	37 (16.02%)	53 (12.44%)	11 (6.88%)	40 (20.51%)	51 (14.37%)	104 (13.32%)
Unspecified education	13 (6.67%)	6 (2.60%)	19 (4.46%)	13 (8.13%)	11 (5.64%)	24 (6.76%)	43 (5.51%)
Employment status							
Unemployed	87 (44.62%)	90 (38.96%)	177 (41.55%)	72 (45.00%)	80 (41.03%)	152 (42.82%)	329 (42.13%)
Employed	42 (21.54%)	46 (19.91%)	88 (20.66%)	38 (23.75%)	39 (20.00%)	77 (21.69%)	165 (21.13%)
Student	1 (0.51%)	3 (1.30%)	4 (0.94%)	4 (2.50%)	3 (1.54%)	7 (1.97%)	11 (1.41%)
Retired	65 (33.33%)	89 (38.53%)	154 (36.15%)	45 (28.13%)	72 (36.92%)	117 (32.96%)	271 (34.70%)
Other income	-	3 (1.30%)	3 (0.70%)	1 (0.63%)	1 (0.51%)	2 (0.56%)	5 (0.64%)
Insurance							
Uninsured	34 (17.44%)	23 (9.96%)	57 (13.38%)	27 (16.88%)	16 (8.21%)	43 (12.11%)	100 (12.80%)
Insured	161 (82.56%)	208 (90.04%)	369 (86.62%)	133 (83.13%)	179 (91.79%)	312 (87.89%)	681 (87.20%)
Residence type							
Urban	115 (58.97%)	171 (74.03%)	286 (67.14%)	113 (70.63%)	146 (74.87%)	259 (72.96%)	545 (69.78%)
Rural	80 (41.03%)	60 (25.97%)	140 (32.86%)	47 (29.38%)	49 (25.13%)	96 (27.04%)	236 (30.22%)
Total	195 (100%)	231 (100%)	426 (100%)	160 (100%)	195 (100%)	355 (100%)	781 (100%)

(%)^a^ = count^a^ / Total for column (pre-pandemic, Informed Consent – Signed- count^as^ /Refused- count^ar^) × 100. (%)^b^ = count^b^ / Total for column (pandèmic, Informed Consent – Signed- count^bs^ /Refused- count^br^) × 100. (%)^c^ = count^c^ / 781 × 100, where: count^c^ = count^a^ + count^b^ (total for row).

#### Age group and ICRR

The distribution of ICRR varied across age groups, with older patients being more likely to have their admission confirmed. ICRR generally decreased with age up to the 40–49 age group, with those under 20 years refusing at 40.00% (4/10) vs. 36.36% (4/11), followed by the 20–29 group at 61.11% (55/90) vs. 58.21% (39/67) and the 30–39 group at 58.65% (61/104) vs. 58.82% (40/68). Patients aged 40–49 confirmed at 53.61% (52/97) vs. 48.75% (39/80), while those aged 50–59 showed 37.74% (20/53) vs. 59.46% (44/74). Among patients 60 years and older, rates were 54.17% (39/72) vs. 52.73% (29/55). However, age was not a statistically significant predictor across periods (pre-vs pandemic), and the association remained weak and non-significant (Chi-Square *p* = 0.0985 vs. *p* = 0.5500, Cramer’s V 0.1584 vs. 0.1181). Controlling for Group Study, the CMH test (*p* = 0.2212) further confirmed that age did not significantly influence ICRR across study periods.

#### Insurance status and ICRR

Insurance status was significantly associated with ICRR, with notable differences between the pre-pandemic and pandemic periods. ICRR in insured patients increased (56.37% [208/369] to 57.37% [179/312]), while in uninsured patients decreased (40.35% [23/57] to 37.21% [16/43]). Albeit statistically significant, the association was weak to small-moderate (Chi-Square *p* = 0.0239 vs. *p* = 0.0127, Cramer’s V = 0.1095 vs. 0.1322), suggesting a slightly stronger relationship in the pandemic period. Controlling for Group_Study, the CMH test (*p* = 0.0008) confirmed a significant association between insurance status and ICRR across both periods, and this relationship’s strength remained stable over time (Breslow-Day, *p* = 0.6958).

#### Marital status and ICRR

Marital status was not significantly associated with ICRR before the pandemic but became significant during the pandemic. Single patients had higher ICRR in both periods (54.55% [60/110]vs. 61.25% [49/80]), and married patients had a slight decrease (61.36% [54/88] vs. 60.81% [45/74]). Divorced patients had a lower ICRR (63.64% [28/44] vs. 73.08% [19/26]), while widowed patients showed minimal change (60.00% [12/20] vs. 63.64% [7/11]). Patients with unspecified marital status had the lowest proportions in both periods, with ICRR at 46.95% (77/164) vs. 45.73% (75/164). Statistical tests showed a weak and non-significant association pre-pandemic (Chi-Square *p* = 0.1284, Cramer’s V = 0.1295) becoming significant with a moderate effect during the pandemic (Chi-Square *p* = 0.0195, Cramer’s V = 0.1817). The association across both periods was significant (CMH, *p* = 0.0018).

#### Level of education and ICRR

Education level was significantly associated with ICRR in both study periods, with higher education levels correlating with higher ICRR. No formal education had the lowest prepandemic ICRR (10.53%, 2/19), increasing to (38.71%, 12/31), followed by unspecified education (31.58%, 6/19 vs. 45.83%, 11/24). Vocational education had moderate ICRR (43.64%, 24/55 vs. 39.47%, 15/38), while primary education slightly decreased (50.00%, 49/98 vs. 43.75%, 35/80). Middle school education had a higher ICRR (52.69%, 49/93 vs. 57.63%, 34/59), whereas higher education had one of the highest ICRRs (69.81%, 37/53 pre-pandemic vs. 78.43%, 40/51 pandemic). High school graduates consistently had the highest ICRR (71.91%, 64/89 vs. 66.67%, 48/72). Statistical tests confirmed a strong association (Chi-Square *p* < 0.0001 vs. *p* = 0.0001, Cramer’s V 0.2995 vs. 0.2776), with the CMH test (*p* = 0.0011) confirming consistency across both periods.

#### Residence type and ICRR

Residence type was significantly associated with ICRR before the pandemic but weakened during the pandemic. Urban patients constituted 67.14% (286/426) pre-pandemic and 72.96% (259/355) during the pandemic, with ICRR decreasing from 59.79% (171/286) to 56.37% (146/259). Rural patients accounted for 32.86% (140/426) pre-pandemic and 27.04% (96/355) during the pandemic, with ICRR increasing from 42.86% (60/140) to 51.04% (49/96). The association was significant pre-pandemic (Chi-Square *p* = 0.0010, Cramer’s V = 0.1596), indicating a small-to-moderate effect size, but became non-significant during the pandemic (Chi-Square *p* = 0.3701, Cramer’s V = 0.0476). Statistical tests confirmed that the association remained stable over time (CMH test *p* = 0.0021, Breslow-Day test *p* = 0.1391).

#### Income source and ICRR

Employment status did not significantly impact ICRR in either period. Unemployed individuals comprised 41.55% (*n* = 177) pre-pandemic and 42.82% (*n* = 152) pandemic, with an ICRR of 50.85% (*n* = 90) vs. 52.63% (*n* = 80). Employed patients accounted for 20.66% (*n* = 88) vs. 21.69% (*n* = 77), with ICRR of 52.27% (*n* = 46) vs. 50.65% (*n* = 39). Retired individuals represented 36.15% (*n* = 154) vs. 32.96% (*n* = 117), refusing consent at 57.79% (*n* = 89) vs. 61.54% (*n* = 72). Students were 0.94% (*n* = 4) pre-pandemic and 1.97% (*n* = 7) pandemic, with confirmation rates of 75.00% (*n* = 3) vs. 42.86% (*n* = 3). Other income sources accounted for 0.70% (*n* = 3) vs. 0.56% (*n* = 2), refusing to consent 100% (3/3) vs. 50% (1/2).

Statistical tests indicated a weak to moderate and non-significant association (Chi-Square *p* = 0.2908 pre-pandemic, *p* = 0.4948 pandemic; Cramer’s V 0.1080 vs. 0.0977). The CMH test (*p* = 0.0329).

### Clinical and psychiatric symptoms

#### Psychotic symptoms and ICRR

Psychotic symptoms at admittance strongly influenced ICRR in both periods (pre-pandemic vs. pandemic; Table 2). Patients with psychotic symptoms had higher ICRR (69.65%, 179/257 vs. 69.92%,165/236) while those without had lower ICRR (30.77%, [52/169] vs. pandemic 25.21%, [30/119]). The association was substantial and statistically significant pre-pandemic (Chi-Square *p* < 0.0001, Cramer’s V 0.3818) and became even stronger during the pandemic (*p* < 0.0001, Cramer’s V 0.4241). The relationship remained stable over time (CMH [*p* < 0.0001], Breslow-Day [*p* = 0.3851]), suggesting that psychotic symptoms remained a strong and consistent predictor of ICRR, unaffected by pandemic-related factors.

**Table 2:** Distribution of informed consent refusal rates (ICRR) by clinical and psychiatric factors across pre-pandemic and pandemic periods.

Clinical and psychiatric symptoms	Group study						Total
	Pre-pandemic			Pandemic			
	Informed consent		Total	Informed consent		Total	
	Signed	Refused		Signed	Refused		
	Count^as^ (%)^as^	Count^ar^ (%)^ar^	Count^a^ (%)^a^	Count^bs^ (%)^bs^	Count^br^ (%)^br^	Count^b^ (%)^b^	Count^c^ (%)^c^
Psychotic symptoms							
No	117 (60.00%)	52 (22.51%)	169 (39.67%)	89 (55.63%)	30 (15.38%)	119 (33.52%)	288 (36.88%)
Yes	78 (40.00%)	179 (77.49%)	257 (60.33%)	71 (44.38%)	165 (84.62%)	236 (66.48%)	493 (63.12%)
Aggressive behavior							
No	55 (28.21%)	110 (47.62%)	165 (38.73%)	36 (22.50%)	82 (42.05%)	118 (33.24%)	283 (36.24%)
Yes	140 (71.79%)	121 (52.38%)	261 (61.27%)	124 (77.50%)	113 (57.95%)	237 (66.76%)	498 (63.76%)
Suicidal behavior							
No	169 (86.67%)	214 (92.94%)	383 (89.91%)	136 (85.00%)	178 (91.28%)	314 (88.45%)	697 (89.24%)
Yes	26 (13.33%)	17 (7.36%)	43 (10.09%)	24 (15.00%)	17 (8.72%)	41 (11.55%)	84 (10.76%)
Psychomotor agitation							
No	36 (18.46%)	68 (29.44%)	104 (24.41%)	28 (17.50%)	38 (19.49%)	66 (18.59%)	170 (21.77%)
Yes	159 (81.54%)	163 (70.56%)	322 (75.59%)	132 (82.50%)	157 (80.51%)	289 (81.41%)	611 (78.23%)
Primary diagnosis							
F00-F09	25 (12.82%)	22 (9.52%)	47 (11.03%)	15 (9.38%)	9 (4.62%)	24 (6.76%)	71 (9.09%)
F10-F19	55 (28.21%)	14 (6.06%)	69 (16.20%)	58 (36.25%)	8 (4.10%)	66 (18.59%)	135 (17.29%)
F20-F29	47 (24.10%)	123 (53.25%)	170 (39.91%)	38 (23.75%)	118 (60.51%)	156 (43.94%)	326 (41.74%)
F30-F31	13 (6.67%)	45 (19.48%)	58 (13.62%)	14 (8.75%)	43 (22.05%)	57 (16.06%)	115 (14.72%)
F32-39	5 (2.56%)	4 (1.73%)	9 (2.11%)	7 (4.38%)	2 (1.03%)	9 (2.54%)	18 (2.30%)
F60-F69	40 (20.51%)	19 (8.23%)	59 (13.85%)	24 (15.00%)	12 (6.15%)	36 (10.14%)	95 (12.16%)
Others	10 (5.13%)	4 (1.73%)	14 (3.29%)	4 (2.50%)	3 (1.54%)	7 (1.97%)	21 (2.69%)
Comorbidities							
Without	96 (49.23%)	164 (71.00%)	260 (61.03%)	71 (44.38%)	141 (72.31%)	212 (59.72%)	472 (60.44%)
With	99 (50.77%)	67 (29.00%)	166 (38.97%)	89 (55.63%)	54 (27.69%)	143 (40.28%)	309 (39.56%)
Total	195 (100%)	231 (100%)	426 (100%)	160 (100%)	195 (100%)	355 (100%)	781 (100%)

(%)^a^ = count^a^ / Total for column (pre-pandemic, Informed Consent – Signed- count^as^ /Refused- count^ar^) × 100. (%)^b^ = count^b^ / Total for column (pandèmic, Informed Consent – Signed- count^bs^ /Refused- count^br^) × 100; (%)^c^ = count^c^ / 781 × 100, where: count^c^ = count^a^ + count^b^ (total for row).

#### Aggressive behavior and ICRR

Aggressive behavior was significantly associated with ICRR across both periods. The proportion of patients exhibiting aggressive behavior increased from 61.27% (261/426) pre-pandemic to 66.76% (237/355) during the pandemic, with the ICRR slightly rising from 46.36% (121/261) to 47.68% (113/237). Non-aggressive cases decreased from 38.73% (165/426) to 33.24% (118/355), nevertheless their ICRR slightly increased too (66.67% [110/165] pre-pandemic to 69.49% [82/118] pandemic). Statistical tests confirmed a small to moderate and stable association. Chi-square tests were significant pre-pandemic (*p* < 0.0001, Cramer’s V = 0.1986) and during the pandemic (*p* < 0.0001, Cramer’s V = 0.2065). Controlling for the Study_Group, the Mantel–Haenszel test (*p* < 0.0001) reinforced the association. Non-aggressive individuals were likelier to have their informed consent refused despite the higher prevalence of aggression among admitted patients [Mantel–Haenszel odds ratio (0.418, 95% CI: 0.3078–0.5676)]. This relationship remained consistent over time (Breslow-Day test [*p* = 0.8070]).

#### Suicidal behavior and ICRR

Suicidal behavior showed a weak association with informed consent refusal, with slightly higher ICRR in both study periods. The majority of patients did not exhibit suicidal behavior (89.91% [383/426] vs. 88.45% [314/355]), with ICRR at 55.87% (214/383) vs. 56.69% (178/314). Before the pandemic, 10.09% (43/426) of patients exhibited suicidal behavior, increasing slightly to 11.55% (41/355) during the pandemic. Among suicidal patients, ICRR was 39.53% (17/43) vs. 41.46% (17/41). Statistical tests confirmed a weak but stable association between suicidal behavior and ICRR. Chi-square tests were significant pre-pandemic (*p* = 0.0414, Cramer’s V = 0.0988) but only marginally substantial during the pandemic (*p* = 0.0654, Cramer’s V = 0.0978). Controlling for the Study Group, suicidal behavior patients had lower odds of ICRR compared to non-suicidal patients (Mantel–Haenszel odds ratio [0.5283, 95% CI: 0.3332–0.8375], *p* = 0.0061). This relationship remained stable over time (Breslow-Day test [*p* = 0.9204]), suggesting no significant change in how suicidal behavior influenced informed consent decisions during the pandemic.

#### Psychomotor agitation and ICRR

Before the pandemic, 75.59% (322/426) of involuntary admissions involved patients with psychomotor agitation, increasing to 81.41% (289/355) during the pandemic, while those without agitation decreased from 24.41% (104/426) to 18.59% (66/355). Among patients without agitation, ICRR decreased from 65.38% (68/104) pre-pandemic to 57.58% (38/66) during the pandemic. In contrast, among those with agitation, ICRR increased from 50.62% (163/322) pre-pandemic to 54.33% (157/289) during the pandemic. Statistical analysis confirmed that before the pandemic, psychomotor agitation was significantly associated with ICRR (Chi-Square *p* = 0.0086, Cramer’s V = 0.1273), suggesting that agitation influenced refusal rates during this period. However, this association weakened and became non-significant during the pandemic (Chi-Square *p* = 0.6320, Cramer’s V = 0.0254), indicating that psychomotor agitation was no longer a significant factor in refusal decisions.

#### Primary diagnosis and ICRR

Schizophrenia spectrum disorders (F20-F29) remained the most prevalent diagnosis, increasing from 39.91% (170/426) pre-pandemic to 43.94% (156/355) pandemic, with ICRR of 72.35% (123/170) pre-pandemic vs. 75.64% (118/156) pandemic. Bipolar affective disorders (F30-F31) rose from 13.62% (58/426) to 16.06% (57/355), with ICRR at 77.59% (45/58) pre-pandemic vs. 75.44% (43/57) pandemic. Substance-related disorders (F10-F19) increased from 16.20% (69/426) to 18.59% (66/355), with ICRR decreasing from 20.29% (14/69) to 12.12% (8/66). Neurocognitive disorders (F00-F09) declined from 11.03% (47/426) to 6.76% (24/355), with ICRR of 46.81% (22/47) pre-pandemic vs. 37.50% (9/24) pandemic. Personality and behavior disorders (F60-F69) decreased from 13.55% (59/426) to 10.14% (36/355), with a slight increase in ICRR: 32.20% (19/59) pre-pandemic vs. 33.33% (12/36) pandemic. The percentage of people diagnosed with other affective disorders (F32-F39) remained relatively constant over the two periods: 2.11% (9/426) pre-pandemic vs. 2.54% (9/355) pandemic, with an ICRR of 44.44% (4/9) vs. 22.22% (2/9). The other mental disorders, which do not fall into the categories mentioned above, have decreased during the pandemic period (3.29% [14/426] vs. 1.97% [7/355]), with an increase of ICRR 28.57% (4/14) pre-pandemic vs. 42.86% (3/7). Statistical analysis confirmed a strong association between primary diagnosis and ICRR, with Chi-Square tests showing significance (*p* < 0.0001 pre-pandemic, Cramer’s V = 0.4908 vs. *p* < 0.0001 pandemic, Cramer’s V = 0.5297), and the Cochran–Mantel–Haenszel (CMH) test (*p* < 0.0001) reinforcing that primary diagnosis consistently influenced ICRR across both periods.

#### Comorbidities and ICRR

The psychiatric comorbidities significantly influenced ICRR in both periods. Before the pandemic, 38.97% (166/426) of patients had at least one comorbidity, increasing slightly to 40.28% (143/355) during the pandemic. Among patients without comorbidities, ICRR were 63.08% (164/260) pre-pandemic vs. 66.51% (141/212) pandemic, while those with comorbidities had lower ICRR [40.36% (67/166) vs. 37.76% (54/143)].

Statistical tests confirmed a stable but significant association (Chi-Square *p* < 0.0001 pre- vs. pandemic) with a weak-to-moderate effect (Cramer’s V −0.2224 pre-pandemic, −0.2834 pandemic). When controlling for group study, patients with comorbidities had significantly lower odds of ICRR (CMH *p* < 0.0001, OR = 0.3525, 95% CI: 0.2622–0.4741). The Breslow-Day test (*p* = 0.3928) indicated no significant shift in association over time, implying that pandemic-related factors did not alter the influence of comorbidities on IA decisions.

### Behavioral factors and substance use

#### Alcohol consumption and ICRR

Alcohol consumers accounted for 34.51% (147/426) pre-pandemic and 34.08% (121/355) during the pandemic, with ICRR decreasing from 31.29% (46/147) to 20.66% (25/121; Table 3). Non-drinkers made up 65.49% (279/426) pre-pandemic and 65.92% (234/355) during the pandemic, with ICRR increasing from 66.31% (185/279) to 72.65% (170/234). Statistical tests confirmed a moderate-to-strong inverse relationship between alcohol consumption and ICRR, with a significant effect pre-pandemic (Chi-Square *p* < 0.0001, Cramer’s V = 0.3341) and a stronger effect during the pandemic (Chi-Square *p* < 0.0001, Cramer’s V = 0.4953). Alcohol consumers were more likely to provide informed consent compared to non-drinkers (CMH test *p* < 0.0001, OR = 0.1632, 95% CI: 0.1175–0.2266) and the effect of alcohol consumption on ICRR differed across periods (Breslow-Day test, *p* = 0.0127).

**Table 3:** Distribution of informed consent refusal rates (ICRR) by behavioral and substance use factors across pre-pandemic and pandemic periods.

Substance use and behavioral factors	Group study						Total
	Pre-pandemic			Pandemic			
	Informed consent		Total	Informed consent		Total	
	Signed	Refused		Signed	Refused		
	Count^as^ (%)^as^	Count^ar^ (%)^ar^	Count^a^ (%)^a^	Count^bs^ (%)^bs^	Count^br^ (%)^br^	Count^b^ (%)^b^	Count^c^ (%)^c^
Alcohol consumption							
No	94 (48.21%)	185 (80.09%)	279 (65.49%)	64 (40.00%)	170 (87.18%)	234 (65.92%)	513 (65.69%)
Yes	101 (51.79%)	46 (19.91%)	147 (34.51%)	96 (60.00%)	25 (12.82%)	121 (34.08%)	268 (34.31%)
Psychoactive substance use							
No	176 (90.26%)	204 (88.31%)	380 (89.20%)	148 (92.50%)	179 (91.79%)	327 (92.11%)	707 (90.52%)
Yes	19 (9.74%)	27 (11.69%)	46 (10.80%)	12 (7.50%)	16 (8.21%)	28 (7.89%)	74 (9.48%)
First-time admission							
No	113 (57.95%)	141 (61.04%)	254 (59.62%)	83 (51.88%)	113 (57.95%)	196 (55.21%)	450 (57.62%)
Yes	82 (42.05%)	90 (38.96%)	172 (40.38%)	77 (48.13%)	82 (42.05%)	159 (44.79%)	331 (42.38%)
Total	195 (100%)	231 (100%)	426 (100%)	160 (100%)	195 (100%)	355 (100%)	781 (100%)

(%)^a^ = count^a^ / Total for column (pre-pandemic, Informed Consent – Signed- count^as^ /Refused- count^ar^) × 100. (%)^b^ = count^b^ / Total for column (pandèmic, Informed Consent – Signed- count^bs^ /Refused- count^br^) × 100; (%)^c^ = count^c^ / 781 × 100, where: count^c^ = count^a^ + count^b^ (total for row).

#### Psychoactive substance use and ICRR

Substance users comprised 10.80% (46/426) pre-pandemic and 7.89% (28/355) during the pandemic, with ICRR slightly decreasing from 58.70% (27/46) to 57.14% (16/28). Non-users accounted for 89.20% (380/426) pre-pandemic and 92.11% (327/355) during the pandemic, with ICRR remaining stable at 53.68% (204/380) vs. 54.74% (179/327). Statistical tests confirmed no significant association between psychoactive substance use and ICRR, with non-significant Chi-Square results pre-pandemic (*p* = 0.5194, Cramer’s V = 0.0312) and during the pandemic (*p* = 0.8063, Cramer’s V = 0.0130). The CMH test (*p* = 0.5115) reinforced this conclusion, while the Breslow-Day test (*p* = 0.8344) revealed a stable relationship over time.

#### First-time admission and ICRR

First-time admissions accounted for 40.38% (172/426) pre-pandemic and increased slightly to 44.79% (159/355) during the pandemic, with ICRR remaining stable at 52.33% (90/172) vs. 51.57% (82/159). Previously hospitalized patients comprised 59.62% (254/426) pre-pandemic and 55.21% (196/355) during the pandemic, with ICRR at 55.51% (141/254) vs. 57.65% (113/196). Statistical analysis confirmed no significant association between first-time admission status and ICRR, with non-significant Chi-Square results pre-pandemic (*p* = 0.5172, Cramer’s V = 0.0314) and during the pandemic (*p* = 0.2522, Cramer’s V = 0.0608). This association was reinforced by the CMH test (*p* = 0.2109), which remained stable across both periods while the Breslow-Day test (*p* = 0.6879) indicated that the relationship remained stable across both periods.

### Legal and admission circumstances

#### Police involvement and ICRR

Patients not brought in by law enforcement comprised 38.03% (162/426) pre-pandemic and 35.77% (127/355) during the pandemic, with ICRR increasing from 62.35% (101/162) to 67.72% (86/127; Table 4). Among police-referred patients, who made up 61.97% (264/426) pre-pandemic and 64.23% (228/355) during the pandemic, ICRR remained lower at 49.24% (130/264) vs. 47.81% (109/228). Statistical analysis confirmed a significant and stable inverse association between police involvement and ICRR, with a stronger effect during the pandemic (Chi-Square *p* = 0.0084, Cramer’s V = 0.1277 pre-pandemic; *p* = 0.0003, Cramer’s V = 0.1918 pandemic). Police-referred patients had significantly lower odds of ICRR compared to non-police cases (CMH test *p* < 0.0001, OR = 0.5149, 95% CI: 0.3818–0.6945), while the Breslow-Day test (*p* = 0.3401) confirmed that this association remained stable across both periods.

**Table 4:** Distribution of informed consent refusal rates (ICRR) by legal and admission circumstances across pre-pandemic and pandemic periods.

Legal and admission circumstances	Group study						Total
	Pre-pandemic			Pandemic			
	Informed consent		Total	Informed consent		Total	
	Signed	Refused		Signed	Refused		
	Count^as^ (%)^as^	Count^ar^ (%)^ar^	Count^a^ (%)^a^	Count^bs^ (%)^bs^	Count^br^ (%)^br^	Count^b^ (%)^b^	Count^c^ (%)^c^
Police involvement							
No	61 (31.28%)	101 (43.72%)	162 (38.03%)	41 (25.63%)	86 (44.10%)	127 (35.77%)	289 (37.00%)
Yes	134 (68.72%)	130 (56.28%)	264 (61.97%)	119 (74.38%)	109 (55.90%)	228 (64.23%)	492 (63.00%)
Involuntary admission request source							
Parents	38 (19.49%)	65 (28.14%)	103 (24.18%)	26 (16.25%)	52 (26.67%)	78 (21.97%)	181 (23.18%)
Children	20 (10.26%)	19 (22.62%)	39 (9.15%)	16 (10.00%)	29 (14.87%)	45 (12.68%)	84 (10.76%)
Sibling	13 (6.67%)	26 (11.26%)	39 (9.15%)	11 (6.88%)	17 (8.72%)	28 (7.89%)	67 (8.58%)
Spouse	21 (10.77%)	24 (10.39%)	45 (10.56%)	7 (4.38%)	14 (7.18%)	21 (5.92%)	66 (8.45%)
Doctor	4 (2.05%)	27 (11.69%)	31 (7.28%)	15 (9.38%)	24 (12.31%)	39 (10.99%)	70 (8.96%)
Police	99 (50.77%)	70 (30.30%)	169 (39.67%)	85 (53.13%)	59 (30.26%)	144 (40.56%)	313 (40.08%)
Recurrence of admission							
No	152 (77.95%)	178 (77.06%)	330 (77.46%)	134 (83.75%)	159 (81.54%)	293 (82.54%)	623 (79.77%)
Yes	43 (22.05%)	53 (22.94%)	96 (22.54%)	26 (16.25%)	36 (18.46%)	62 (17.46%)	158 (20.23%)
Total	195 (100%)	231 (100%)	426 (100%)	160 (100%)	195 (100%)	355 (100%)	781 (100%)

(%)^a^ = count^a^ / Total for column (pre-pandemic, Informed Consent – Signed- count^as^ /Refused- count^ar^) × 100. (%)^b^ = count^b^ / Total for column (pandèmic, Informed Consent – Signed- count^bs^ /Refused- count^br^) × 100; (%)^c^ = count^c^ / 781 × 100, where: count^c^ = count^a^ + count^b^ (total for row).

#### IA request source and ICRR

During the pandemic, the ICRR increased mainly among children-initiated referrals, rising from 48.72% (19/39) pre-pandemic to 64.44% (29/45). Spouse-initiated refusals also increased, from 53.33% (24/45) to 66.67% (14/21), while the ICRR in the cases of patients with Involuntary Admission Request by parents has increased from 63.11% (65/103) to 66.67% (52/78). In contrast, doctor-initiated ICRR significantly decreased, from 87.10% (27/31) pre-pandemic to 61.54% (24/39). Police-initiated refusals remained relatively stable, showing a slight decrease from 41.42% (70/169) to 40.97% (59/144), while sibling-initiated refusals declined from 66.67% (26/39) to 60.71% (17/28). Statistical analysis confirmed a significant association between referral source and ICRR in both periods, with Chi-Square tests yielding *p* < 0.0001 pre-pandemic (Cramer’s V = 0.2691) and *p* = 0.0015 during the pandemic (Cramer’s V = 0.2347). The CMH test (*p* < 0.0001) reinforced a strong association between referral source and ICRR.

#### Readmission and ICRR

Before the pandemic, patients hospitalized involuntarily for the first time made up 77.46% (330/426), increasing to 82.54% (293/355) during the pandemic. Among them, ICRR remained stable at 53.94% (178/330) pre-pandemic vs. 54.27% (159/293) during the pandemic. Readmitted patients accounted for 22.54% (96/426) pre-pandemic and 17.46% (62/355) during the pandemic, with ICRR showing minimal change at 55.21% (53/96) vs. 58.06% (36/62). Statistical analysis confirmed no significant association between readmission and ICRR in either period (Chi-Square *p* = 0.5850, Cramer’s V = 0.0290, CMH *p* = 0.6053). The Mantel–Haenszel odds ratio (OR = 1.0975, 95% CI: 0.7715–1.5612) indicated that hospitalization history did not influence the likelihood of ICRR. The Breslow-Day test (*p* = 0.7783) confirmed that this relationship remained stable.

### Restraint and timing factors

#### Physical restraint and ICRR

The proportions of patients requiring restraint increased from 32.63% (139/426) to 36.90% (131/355) with ICRR slightly rising from 52.52% (73/139) to 54.20% (71/131; Table 5). The number of patients who did not require physical restraint decreased from 67.37% (287/426) pre-pandemic to 63.10% (224/355) during the pandemic with ICRR remaining stable at 55.05% (158/287) pre-pandemic vs. 55.36% (124/224) during the pandemic. Statistical analysis confirmed no significant association between physical restraint and ICRR in either period (pre-pandemic: Chi-Square *p* = 0.6225, Cramer’s V = 0.0238); pandemic: Chi-Square (*p* = 0.8323, Cramer’s V = 0.0112), while the Breslow-Day test (*p* = 0.8554) showed that the association remained stable over time.

**Table 5:** Distribution of informed consent refusal rates (ICRR) by restraint use and admission timing across pre-pandemic and pandemic periods.

Restraint and timing factors	Group study						Total
	Pre-pandemic			Pandemic			
	Informed consent		Total	Informed consent		Total	
	Signed	Refused		Signed	Refused		
	Count^as^ (%)^as^	Count^ar^ (%)^ar^	Count^a^ (%)^a^	Count^bs^ (%)^bs^	Count^br^ (%)^br^	Count^b^ (%)^b^	Count^c^ (%)^c^
Physical restraint used							
Not required	129 (66.15%)	158 (68.40%)	287 (67.37%)	100 (62.50%)	124 (63.59%)	224 (63.10%)	511 (65.43%)
Required	66 (33.85%)	73 (31.60%)	139 (32.63%)	60 (37.50%)	71 (36.41%)	131 (36.90%)	270 (34.57%)
Admission time							
Day	115 (58.97%)	147 (63.64%)	262 (61.50%)	91 (56.88%)	127 (65.13%)	218 (61.41%)	480 (61.46%)
Night	80 (41.03%)	84 (36.36%)	164 (38.50%)	69 (43.13)	68 (34.87%)	137 (38.59%)	301 (38.54%)
Total	195 (100%)	231 (100%)	426 (100%)	160 (100%)	195 (100%)	355 (100%)	781 (100%)

(%)^a^ = count^a^ / Total for column (pre-pandemic, Informed Consent – Signed- count^as^ /Refused- count^ar^) × 100. (%)^b^ = count^b^ / Total for column (pandèmic, Informed Consent – Signed- count^bs^ /Refused- count^br^) × 100; (%)^c^ = count^c^ / 781 × 100, where: count^c^ = count^a^ + count^b^ (total for row).

#### Admission time and ICRR

The proportion of daytime admissions remained consistent, with 61.50% (262/426) pre-pandemic and 61.41% (218/355) during the pandemic, ICRR increasing slightly from 56.11% (147/262) to 58.26% (127/218). Nighttime admissions also showed stability at 38.50% (164/426) pre-pandemic and 38.59% (137/355) during the pandemic with ICRR remaining nearly unchanged at 51.22% (84/164) pre-pandemic and 49.64% (68/137) during the pandemic. Statistical analysis confirmed no significant association between admission timing and ICRR in either period (pre-pandemic: Chi-Square *p* = 0.3245, Cramer’s V = 0.0477; pandemic: Chi-Square *p* = 0.1120, Cramer’s V = 0.0844). The relationship remained stable across both periods (Breslow-Day test, *p* = 0.6101), suggesting that external crises, such as pandemic-related stressors, did not significantly influence when patients were admitted involuntarily.

### Machine learning model

**Figure 1:**
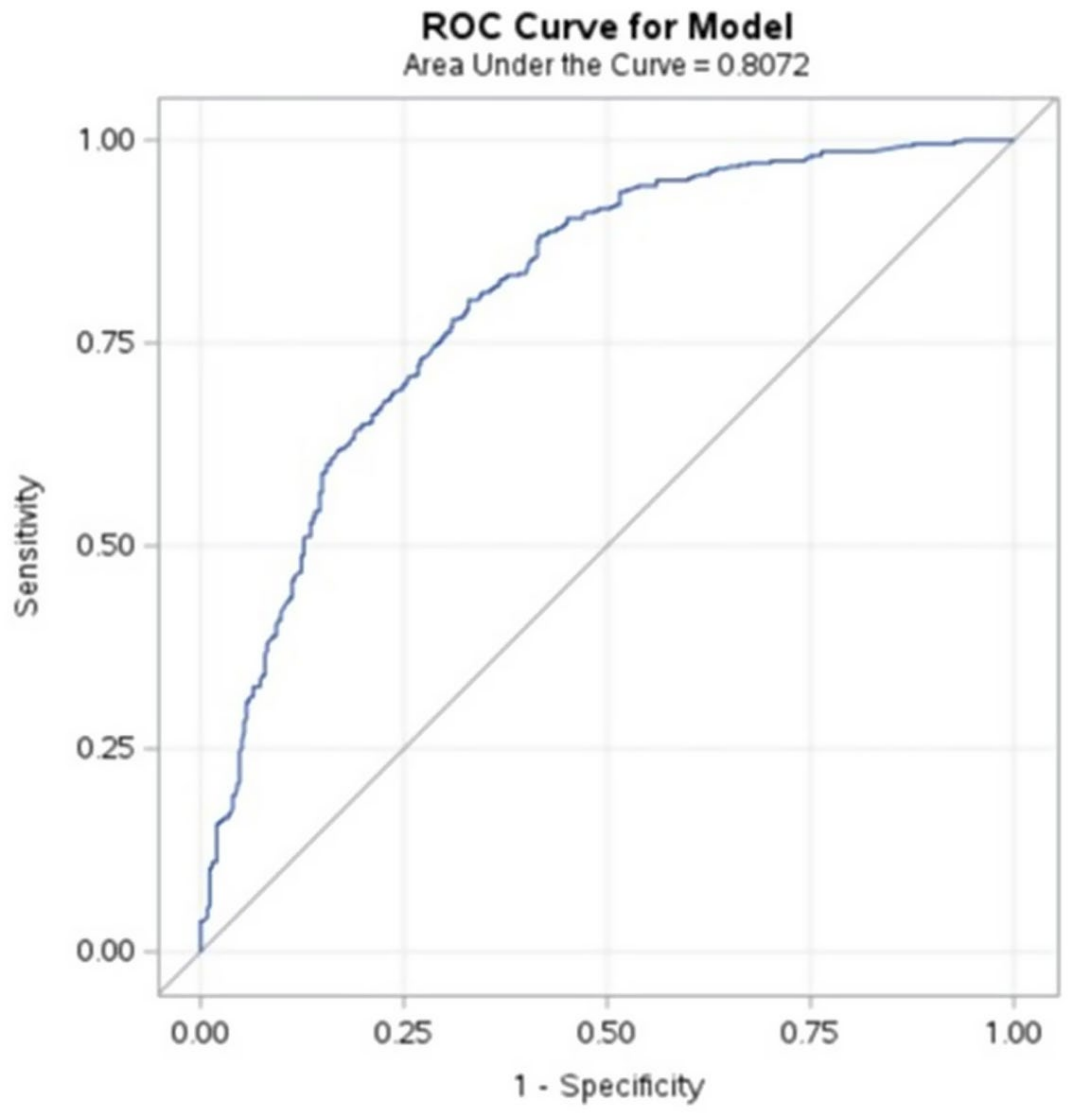
The receiver operating characteristic (ROC) curve of the logistic regression model plotting sensitivity (true positive rate) against 1—specificity (false positive rate) at various threshold settings.

The logistic regression model predicted the likelihood of ICRR = 1 based on the key sociodemographic, clinical, and behavioral factors that demonstrated statistical significance and were consistently supported across all preliminary analyses. Specifically, we retained for our final regression model education level, alcohol consumption, substance disorders, and police-initiated admissions based on Wald χ^2^ (*p* < 0.05). The model (Equation 1) showed strong predictive ability (AUC = 0.807) and statistical significance (Likelihood Ratio χ^2^ = 242.56, *p* < 0.0001), while exhibiting good calibration (Hosmer–Lemeshow χ^2^(8) = 12.59, *p* = 0.13; Figure 1).


(1) $$\begin{array}{l} \log(\frac{P(\text{ICRR}=1)}{1-P(\text{ICRR}=1)})=-0.8903+1.1625(\text{Primary Education}\;)\\ +1.0179(\text{Middle School})+1.4677\;(\text{High School})\\ +1.4237(\text{Higher Education})-0.5615(\text{Alcohol Consumption})\\ -1.0696(\text{Substance Disorders})\\ -0.6934(\text{Police}-\text{Initiated Admission}) \end{array}$$

Education level emerged as a strong predictor of ICRR, with higher education levels associated with an increased likelihood of refusal. Compared to patients with no formal education, those with higher education were more than four times as likely to refuse consent (OR = 4.153, 95% CI: 1.748–9.862, *p* = 0.0013), while those with high school education were also significantly more likely to refuse (OR = 4.339, 95% CI: 1.949–9.660, *p* = 0.0003). Patients with middle school education (OR = 2.767, 95% CI: 1.249–6.130, *p* = 0.0121) and primary education (OR = 3.198, 95% CI: 1.460–7.006, *p* = 0.0037) also showed significantly higher refusal rates. Alcohol consumption was associated with a significantly lower likelihood of refusal, as alcohol consumers were 43% less likely to refuse consent compared to non-drinkers (OR = 0.570, 95% CI: 0.353–0.921, *p* = 0.0216). Similarly, patients diagnosed with substance-related disorders were 66% less likely to refuse consent (OR = 0.343, 95% CI: 0.164–0.716, *p* = 0.0044), suggesting that individuals with impaired cognitive insight or substance dependency may be more compliant with hospitalization decisions. Police-initiated involuntary admissions were also significantly associated with a lower likelihood of refusal, as patients brought in by law enforcement were 50% less likely to refuse consent (OR = 0.500, 95% CI: 0.294–0.851, *p* = 0.0106) compared to those admitted through parent-initiated referrals. This likely reflects the acute nature of crises requiring law enforcement intervention, where immediate hospitalization is deemed necessary.

## Discussion

To the best of our knowledge, this study offers the first in-depth comparison of IA in Romania before and during the COVID-19 pandemic, revealing significant shifts in patient profiles and admission patterns. Consistent with international reports (24, 41), overall psychiatric admissions declined during the pandemic, while IA cases—particularly involving psychotic disorders—remained frequent or increased (28, 34, 42–44).

**Demographic trends offered additional insights into the impact of the pandemic on IA**. Gender-specific patterns emerged, with female patients showing increased IA and ICRR, and male patients showing decreases partially echoing previous findings (45–47). Age effects were minimal, contrasting with studies that highlight older adults as high-risk (47). Marital status became more relevant during the pandemic: single individuals had higher IA rates, reinforcing prior evidence on social support’s protective role (35, 48). Education level, notably, was associated with increased ICRR during the pandemic, especially among those with higher education. Though this contrasts with some earlier findings (21, 35), it may reflect growing autonomy or mistrust, warranting cautious interpretation and further investigation.

**Geographical and behavioral factors further shaped refusal patterns**. Urban–rural patterns shifted such that while urban patients had higher ICRR pre-pandemic (21), refusal rates rose among rural patients during the pandemic, possibly reflecting healthcare access barriers or changing clinical judgment. Additionally, substance and alcohol use were associated with lower refusal odds, potentially due to symptom remission post-detoxification. This diverges from literature identifying substance use as a risk factor for IA (49–51) suggesting the need for alternative, short-term containment strategies outside the IA framework.

**Institutional dynamics also influenced the course of admissions**. Family-initiated IA requests declined during the pandemic, while police-initiated ones increased. The relationships of dependency and control that may exist between the patient and first-degree relatives, as evidenced in studies of family abuse (52), may pose a risk for manipulation of the decision-making process, using involuntary admission as a tool of coercion. Although police involvement is commonly linked to IA in prior research (53, 54), our data show an association with reduced refusal, possibly reflecting a shift toward voluntary care once crises were managed. Meanwhile, physical restraint use and admission timing remained stable, contrary to other reports (7, 55), suggesting that these procedural elements were less affected by pandemic pressures in our setting.

**Clinical presentation played a critical role in shaping IA outcomes during the pandemic**. Suicidal behavior rose markedly among involuntarily admitted patients (34), aligning with broader literature on pandemic-induced psychological distress (56, 57). These findings underscore the importance of early intervention and sustained psychological support, particularly in crisis contexts (58, 59). Psychomotor agitation remained the leading reason for IA and slightly increased, often co-occurring with aggression—supporting its role as a precursor symptom (56, 60). However, aggression itself was not linked to higher confirmation rates, possibly due to the stabilizing effect of hospitalization (48, 61, 62).

**Diagnostic consistency across the two periods provides additional context**. Schizophrenia-spectrum disorders (F20–F29) remained the most common diagnoses, followed by substance-related (F10–F19) and bipolar disorders (F30–F31), aligning with international data (20, 63). Our findings also mirror Romanian studies showing reduced general admissions but increased IA, particularly for psychotic disorders during lockdown (28). These trends may reflect reduced outpatient access and escalating symptom severity, especially among patients with schizophrenia.

**The machine learning analysis adds further nuance to these findings**. Education level, substance use, and police involvement emerged as significant predictors of ICRR. Unlike prior models (35, 36) we observed a progressive increase in refusal odds with higher education. On the other hand, substance and alcohol use were linked to lower refusal odds, consistent with clinical improvement post-intoxication. Although aggressiveness and certain diagnoses like schizophrenia are established predictors in earlier studies (35, 36), their indirect influence in our model suggests the relevance of context-specific factors and the need for model refinement. The logistic regression model demonstrated robust predictive performance (AUC = 0.807), surpassing previously reported moderate outcomes (AUC = 0.68–0.72) and closely approaching results from advanced machine learning approaches (AUC = 0.84) (35, 36). These comparative insights emphasize the importance of context-specific predictor selection and the potential value of integrating comprehensive clinical, sociodemographic, behavioral, and textual electronic health records (EHR) to enhance predictive accuracy and clinical utility for involuntary psychiatric admissions. Integrated into the EHR, the model would serve as a point-of-intake decision-support tool, flagging patients at high risk of ICRR on the basis of education level, alcohol or substance use, and procedural (police involvement) factors. Targeted interventions such as extended consent discussions, early involvement of patient advocates, or tailored educational materials could then be deployed. Over time, this screening workflow may streamline triage, optimize resource allocation (e.g., assigning more time or specialist staff to high-risk cases), and ultimately reduce coercion by identifying candidates for voluntary engagement before involuntary admission becomes necessary. Prospective validation studies and careful calibration in diverse clinical settings will be essential to minimize false positives and ensure the model complements clinician judgment and patient autonomy.

Overall our findings highlight the complex, multi-layered nature of IA decisions during public health crises—shaped by individual, social, clinical, and institutional dimensions. The limited use of machine learning in this area underscores its potential to inform timely interventions and reduce unnecessary coercion in psychiatric care.

### Strengths and limitations

The present study possesses distinct methodological strengths, yet it is also subject to several limitations. Data collection was conducted retrospectively over 48 months, which could affect the generalizability of the study results. Another limitation of the study is the inclusion of all presentations for an individual with multiple hospitalizations which resulted in duplication of some socio-demographic data. Although this information could reflect changes over the period analyzed (such as marital status, background, insured status, occupation, educational level or age), this was not explicitly controlled for in the analysis. Nevertheless, because the data were anonymized, it was impossible to identify readmitted individuals, assess differences in symptomatology during multiple relapses and include relevant data in this study. Significant variations between health systems and differences in legislation on involuntary admission limit the international comparison of results. Nonetheless, collaborative international studies (21, 35, 46, 64) remain essential to explore the prevalence and characteristics of nonvoluntary hospitalizations and readmissions in diverse settings and cultures, using uniform assessment instruments and standardized methodology. Despite these limitations, the large sample size and the detailed analysis of multiple sociodemographic, clinical and procedural aspects allowed a rigorous and accurate estimation of data from the study population.

Additionally, the machine learning approach employed in our study has limitations when compared to previous reports. Unlike advanced machine learning methods, such as those used by Perfalk et al. (36), our logistic regression model does not inherently account for complex interactions among variables, potentially limiting its predictive accuracy. However, employing interaction terms in logistic regression models would complicate interpretation significantly, making it challenging to translate statistical findings into actionable clinical guidelines. Moreover, the absence of detailed clinical indicators (e.g., aggressiveness scales and specific diagnostic categories) in our analysis, highlighted as significant in prior studies (35, 36) may have constrained our model’s ability to fully capture the multidimensional nature of psychiatric admissions. Nevertheless, including such detailed clinical variables would significantly increase model complexity and potentially diminish practical usability due to more complicated data collection and analysis requirements. Future studies would benefit from balancing detailed clinical information and interpretative clarity with advanced analytical techniques to enhance model performance and clinical applicability.

Furthermore, the single-center design, drawing on data from a single Romanian psychiatric hospital, limits the external validity of both the descriptive findings and the predictive model, given regional differences in admission practices, legal frameworks, and resources. Multicenter studies across diverse healthcare settings are therefore needed to corroborate and extend these results. Accordingly, the predictive algorithm, developed and tested within the same cohort, requires external validation; its robustness and generalizability must be confirmed in independent datasets before wider clinical implementation can be further recommended.

## Conclusion

The COVID-19 pandemic posed significant challenges to health systems worldwide, profoundly affecting psychiatric care and involuntary admissions. In a context marked by severe restrictions, limited resources, and uncertainty, the patterns of involuntary hospitalizations shifted notably, highlighting psychotic symptoms as a key factor in ICRR. Socio-demographic factors, such as gender, education level, and marital status, along with behavioral factors like alcohol use and police involvement, significantly influenced admission outcomes. Predictive modeling further identified education level, alcohol consumption, substance-related disorders, and police-initiated referrals as significant independent predictors of consent refusal, providing valuable insights to anticipate hospitalization needs and inform clinical decision-making. These findings underscore the need for targeted psychiatric interventions and comprehensive crisis management strategies, particularly for vulnerable patient groups. Establishing a unified procedural framework through inter-institutional collaboration between medical, judicial, and law enforcement authorities is essential to address the complex medical and legal aspects of involuntary admissions, ensuring the protection of patients’ fundamental rights.

## Data Availability

The raw data supporting the conclusions of this article will be made available by the first author, upon reasonable request. The code related to this work is available at: https://github.com/bogdanneamtu76/Inv_Admissions.git.

## Appendix A

### The context of Romania

Involuntary admission is regulated in Romania by Law no. 487/2002 on mental health and the protection of persons with mental disorders (31) and Order no. 488/2016 approving the Norms for applying this law (65). Involuntary admission is required when a licensed psychiatrist finds that a person has a mental disorder and, due to it, there is an imminent danger of harm to himself or others or in case of the existence of a severe mental disorder, when his non-admission would cause a serious deterioration of his condition or the impossibility of administering appropriate treatment and only after exhausting the options of voluntary admission (31). In such cases, the patient’s mental capacity is severely impaired, preventing them from realizing the need for these therapeutic and care measures (33). In Europe, two models are used for the establishment of the involuntary admission procedure: the medical model (also practiced in Romania), with the medical procedure prevailing in the case of the measure, where the courts exercise a secondary control role, and the legal model, where the decision to restrict liberty is exclusively a matter for the judges, hence psychiatrists having a secondary role in the application of the involuntary admission procedure (18). According to the current applicable legislation, involuntary admissions can be carried out in psychiatric hospitals and psychiatric wards with adequate conditions for specialized care under specific conditions, the latter being recently introduced by Ordinance No. 18 of January 31, 2024 (32). The involuntary admission procedure begins with the request for involuntary admission, certified under the signature of the holders provided for in Article 55 of Law 487/2002. The next step is the health assessment by the psychiatrist on duty at the psychiatric hospital, who, within 24 h of the evaluation, must send the involuntary admission notification and the related documentation to the involuntary admission committee. The involuntary admissions committee, consisting of two psychiatrists and a doctor of another specialty, considers the proposal of the psychiatrist on duty within 48 h of receipt after examining the person concerned. The decision confirming involuntary admission is then sent to the management of the health facility, which is obliged to send the decision together with the medical documents to the court within 24 h. The court stage is an emergency procedure where the participation and hearing of the patient is mandatory (on-site or online). The court may order confirmation of involuntary admission, in which case the person remains in the hospital. Alternatively, it may be found that involuntary admission is unnecessary and that outpatient treatment should replace it. After the court stage, the involuntary admissions committee is required to re-examine the patient at least once every month, depending on the patient’s condition or at the request of the people entitled to do so (the head doctor, the patient’s legal or conventional representative, the public prosecutor or the patient himself). If the conditions that led to involuntary admission are no longer met, the committee will inform the management of the health unit, which will forward the proposal to terminate involuntary admission to the court, which is the last stage of the process (31).
